# Prevalence of PTSD and anxiety among internally displaced Sudanese children in during war in Al Jabalain White Nile state, Sudan, 2024

**DOI:** 10.1192/j.eurpsy.2025.1126

**Published:** 2025-08-26

**Authors:** E. A. Babiker, E. A. Eisa, M. M. Mohamed Ahmed, S. A. Ibrahim, A. A. Ahmed

**Affiliations:** Medicine, Alneelain University, KHARTOUM, Sudan

## Abstract

**Introduction:**

Sudan is currently experiencing the largest child displacement crisis worldwide. Since the conflict erupted in 2023, over 4.6 million children have been internally displaced within Sudan, and nearly one million are seeking refugee in neighboring countries (UNICEF, 2023). The mental health of these children has been overlooked, and there is a noticeable lack of data on their psychological wellbeing.

**Objectives:**

To assess the prevalence of PTSD, anxiety and factors associated with them among internally displaced Sudanese children during war in IDP camps in Al Jabalain district, White Nile state, Sudan, 2024

**Methods:**

A cross-sectional study was conducted among internally displaced Sudanese children in 11 camps in Al Jabablain district. Children were interviewed using a standerdised questionnaire consisting of the PTSD Civilian Checklist Version 5 abracic version and the Hamilton Scale arabic version to assess the prevalence of PTSD and anxiety. Data analysis was conducted using IBM® SPSS, version 26.0.

**Results:**

The sample included 223 children; age median was 12 years (IQR: 9-15). 127 (57%) of them were females, with majority 128 (57.4%) in primary school. Participants who met the criteria for probable PTSD were 23 (14.3%), (median: 32, IQR: 17-28). 150 (67.3%) participants had mild anxiety, with a median score of 11 (IQR: 9-14). Older children had higher levels of PTSD and anxiety. Also, both scales were significantly associated with gender, displacement frequency and separation from close ones (p value <0.05). Females were more likely suffer from anxiety 116/127(91%) in comparison to males 76/96(79%). Yet, 19(59.4%) of children with probable PTSD were males. Children displaced more than four times were more prone to PTSD symptoms and moderate to severe anxiety. Interestingly, recently displaced children were more likely to have higher PTSD scores and lower anxiety scores. Educational level was significantly associated with PTSD, with about one-third of high school students experienced PTSD symptoms. Anxiety scale was significantly associated with direct exposure to violence, as 13.6% of those exposed to violence experienced mild to moderate anxiety.

**Conclusions:**

This study identified significant levels of PTSD and anxiety among internally displaced children in the Al Jableen camps, highlighting the urgent need for mental health interventions, which promotes resilience and coping skills. Factors such as age, gender, frequency of displacement, direct exposure to violence, and educational level significantly impact the development of these mental health conditions.

**Disclosure of Interest:**

None Declared

